# The Nursing Effect of Individualized Management on Patients With Diabetes Mellitus Type 2 and Hypertension

**DOI:** 10.3389/fendo.2022.846419

**Published:** 2022-03-17

**Authors:** Ran Li, Weiwei Xu, Ping Yang, Lian Tan, Zhiyu Ling, Xiuni Gan

**Affiliations:** ^1^Department of Cardiology, The Second Affiliated Hospital of Chongqing Medical University, Chongqing, China; ^2^Department of Endocrinology and Metabolism, The Second Affiliated Hospital of Chongqing Medical University, Chongqing, China; ^3^Department of Critical Care Medicine, The Second Affiliated Hospital of Chongqing Medical University, Chongqing, China; ^4^Department of Nursing, The Second Affiliated Hospital of Chongqing Medical University, Chongqing, China

**Keywords:** individualized nursing and health education, individualized management, diabetes mellitus type 2, hypertension, rehabilitation efficacy

## Abstract

It focused on clinical effects of individualized nursing and health education (INHE) on patients with diabetes mellitus type 2 (T2DM) and hypertension. 68 patients were randomly rolled into two groups, 34 cases in the control group (group A) received routine nursing and remaining 34 cases in the experimental group (group B) received INHE. The disease knowledge mastery (DKM) and the effect of rehabilitation nursing of patients were compared. The results suggested that DKM of patients in group B was obviously greater (*P* < 0.05). The total effective rate (TER) in group B was 91.45%, which was observably greater than that (76.35%) in group A (*P* < 0.05). After nursing, the fasting plasma glucose (FPG), 2-hour postprandial glucose (2h PG), systolic blood pressure (SBP), and diastolic blood pressure (DBP) levels of all patients decreased, and those in group B were much lower (*P* < 0.05). Scores of the Self-Rating Anxiety Scale (SAS) and Self-Rating Depression Scale (SDS) of the two groups were 56.34 ± 8.12 points and 56.33 ± 8.01 points in group A, respectively; and those in group B were 42.52 ± 6.77 points and 41.71 ± 7.23 points, respectively; and they were all decreased and those in the group B were obviously smaller (*P* < 0.05). In summary, INHE can effectively improve the psychological cognition of patients with T2DM and hypertension and strengthen the control of blood pressure and blood sugar.

## Introduction

As the rapid growth of the world economy and people’s living standards, the dietary structure is changing greatly day by day, and factors such as faster and faster pace of life and lack of exercise have increased the incidence and prevalence of diabetes (1). Diabetes is a public health issue of general concern around the world. It is a lifelong chronic disease that can affect multiple organs; it is the third most common, frequently-occurring, and chronic non-communicable disease after cerebrovascular disease and tumors; and it poses a serious threat to human health (2). Statistics show that there are about 347 million patients suffering from diabetes mellitus type 2 (T2DM) in the world so far, and its prevalence in low-income countries is more serious (3). Statistics from the Chinese Medical Association (CMA) Diabetes Association in 2007 showed that the prevalence of the disease in men and women over 20 years old in China is 10.6% and 8.8%, respectively, and the total prevalence is 9.7%. Based on this estimate, the total number of diabetic patients in China is currently around 92.4 million, and there are 14.82 million patients in the pre-diabetes stage (fasting plasma glucose (FPG) > 6.1 mmol/L, or 2-hour postprandial glucose (2h PG) > 7.8 mmol/L, but they have not yet reached the diagnosis criteria of diabetes). It accounts for nearly 10% and 15% of the total population, which is 4 times the statistical data in 1994 and twice the statistical data in 2001 (4, 5).

T2DM is an endocrine disease, which is very common clinically. It is characterized by high blood sugar, and it is closely related to factors such as weight (obesity) and genetics. In addition, the clinical manifestations are relatively mild at the initial stage of the illness. After the disease worsens, the symptoms of polydipsia, polyphagia, polyuria, and weight loss will occur, which greatly affect the patient’s quality of life (6). Nowadays, evidence-based medicine and clinical studies have shown that prevalence of hypertension in T2DM patients is much greater than that of those without diabetes. In clinical hypertension, it is a very common complication of diabetic patients (7). A report from World Health Organization (WHO) showed that the prevalence of concurrent hypertension in diabetic patients is 20 - 40%, which is about 40 - 50% in China. A surgery from National Health and Nutrition Examination Survey III (NHANES III) found that 71% of 1500 diabetic patients have hypertension at the same time, and the incidence of concurrent hypertension in diabetic patients is 1.5 - 3.0 times that of patients without diabetes. On the contrary, hypertension patients are more likely to develop diabetes than patients with normal blood pressure, and the incidence of diabetes is 2.0 - 2.5 times that of the normal blood pressure group (8, 9). Through epidemiological investigations, it is found that nearly 40% of T2DM patients will have hypertension, and 5% - 10% of primary hypertension may suffer from T2DM (10). These data suggest that T2DM is inseparable from hypertension. The prevention and treatment of diabetes complicated by hypertension has to be solved urgently, so as to improve people’s quality of life and health.

Incidence of hypertension in T2DM patients is increasing, and the patient’s condition will become worse when comorbidities occur. If the symptomatic treatment is not timely, it will cause anxiety and depression symptoms of the patient, and cause damage to the eyes, nerves, heart, and other parts of the patient. What is more, serious comorbidities such as kidney failure, myocardium, and cerebral infarction will occur (11, 12). At the same time, the symptoms of anxiety and depression will in turn affect the patient’s blood sugar and blood pressure control, making the symptoms of anxiety and depression more serious, and then forming a vicious circle. T2DM patients with hypertension have a very obvious curative effect in terms of drug treatment. However, symptoms such as anxiety and depression will seriously affect the occurrence and development of the disease (13).

In the past few years, the general nursing mode such as monitoring the patient’s blood sugar and blood pressure has been mostly used. However, because most diabetic patients are accompanied by hypertension and are prone to some cardiovascular and cerebrovascular diseases, this nursing mode is not effective. Therefore, while actively treating patients, it is particularly important to use a scientific nursing model. The INHE, through scientific guidance on patients’ lifestyle and medication habits, improves the quality of life of patients. For example, it can correct the bad habits of the patient’s diet and ensure a balanced intake of nutrients, so as to effectively control the weight gain, so that the incidence of diabetes is greatly reduced. It can guide patients to carry out reasonable and effective aerobic exercise, help expand the peripheral blood vessels of the body, and reduce peripheral resistance, thereby reducing the occurrence and development of hypertension. There are relatively many clinical studies on the individualized care model or health education for patients with diabetes and hypertension, but relatively few studies on the combination of the two methods (14, 15). Therefore, it explored the clinical effect of the combination of INHE on patients with T2DM and hypertension in this work.

## Research Materials and Methods

### General Data of Included Patients

68 patients with T2DM complicated with hypertension admitted to the Endocrinology Department of our Hospital from March 2018 to February 2020 were rolled into a group B and a group A using the random number table method. 34 cases in the control group (group A) received routine nursing and remaining 34 cases in the experimental group (group B) received INHE. Patients included had to meet the following criteria. First, patients suffered from the symptoms in line with the diagnostic criteria for diabetes defined by World Health Organization (WHO) and complied with the diagnostic criteria for hypertension defined by the *Guidelines for the Prevention and Treatment of Hypertension* in China. Second, the age of the patient was between 34 and 78 years old, and the course of the disease was 3 to 20 years. Third, the measured FPG was ≥ 7.0 mmol/L, the measured 2h PG was ≥ 11.1 mmol/L, the systolic blood pressure (SBP) was ≥ 140 mmHg, and the diastolic blood pressure (DBP) was ≥ 90 mmHg. If following items can be meet, the patients had to be excluded from this study: patients with severe functional disorders of any organ; patients with cancer or secondary diabetes; patients with hearing and speech impairment and dementia; and patients being unable to cooperate and accept the treatment. The general conditions of patients were shown in **Table 1**.

**Table 1:** General data of patients.

	Group B	Group A
Number of cases	34 cases	34 cases
Gender ratio	Males: females = 20:14	Males: females = 23:11
Age	46 ~ 78 years old	43 ~ 74 years old
Average age	56.37 ± 3.56 years old	56.71 ± 3.82 years old
Course of disease	5 ~ 13 years	5 ~ 12 years
Average course of disease	8.02 ± 1.83 years	7.87 ± 1.80 years
FPG	9.73 ± 1.78 mmol/L	9.61 ± 1.72 mmol/L
2h PG	15.53 ± 2.65 mmol/L	15.57 ± 2.64 mmol/L

No statistically obvious difference was found between the two groups of patients in gender, age, course of disease, and other data (*P* > 0.05). The patients and their families in this study had fully understood the situation and signed the informed consent forms. The medical ethics committee of the hospital had known and agreed to implement it.

### Methods of Individualized Management

Patients in group A underwent routine nursing, which referred to routine (admission and diet) guidance, disease introduction, regular monitoring of patients’ FPG and 2h PG levels, and changes in blood pressure. It should monitor the blood sugar and blood pressure control situation of patients, arrange medication plan accordingly, and guide the patient to use medication rationally. Patients in the group B were given INHE on the basis of treatment ways in the group A. The detailed methods were as follows:

The health education was implemented as follows. After the patient was admitted to the hospital, a specific person would be arranged to carry out admission publicity and education to the patient and his family with a kind attitude to tell the patient and his family about the disease-related knowledge, preventive measures, medication plan, emergency management, drug effects and adverse reactions, and exercise plan during the rehabilitation period. The person should give timely and effective responses to patients’ doubts, and regularly distribute materials and play videos to explain relevant knowledge. It should strengthen the awareness of diabetes and confidence in treatment through visits and questionnaire surveys. In this way, it aimed to build a harmonious relationship between doctors and patients and allow patients to actively cooperate with treatment.

The individualized care model was implemented as follows.

First, after the patient was admitted to the hospital, the nurses carefully told the patient and their family about the ward’s environmental equipment, the doctor in charge, and the nurse with a kind attitude, give them more care and nursing, eliminate their bad mood, and make the patient actively cooperate with the treatment.

Second, it can plan the patient’s personal meal plan. For example, it should confirm the total calories required per day and the distribution method of nutrients required for each meal; instruct them to eat less high-cholesterol and high-fat foods, eat more high-potassium and high-calcium foods and more dietary fiber and vitamin content food, avoid drinking black tea, and control the intake of sodium, fat, and carbohydrates.

Third, it should strictly abide by the doctor’s orders, and explain in detail the mechanism of action and adverse reactions of the drug to patients and their families. At the same time, it should inform the patient that metformin drugs should be taken after meals, sulfonylurea hypoglycemic drugs should be taken 30 minutes before meals, and α-glucose anhydrase inhibitors should be taken with the first meal. If symptoms of hypotension (dizziness, nausea, vomiting, etc.) occur, it can slowly move to the supine position with head down and feet up to increase the return to the heart. When paleness, palpitations, and low blood sugar reactions occur, the sugar can be dissolved in warm water and used, and the above reactions can be quickly relieved. If the symptoms are not relieved, they should be sent to a doctor immediately; and the patient and their family members are advised to act mildly to avoid the occurrence of orthostatic hypotension and cerebrovascular disease.

Fourth: it should measure and record blood pressure at 7:00 ~ 8:00 and 19:00 ~ 20:00 every day. When blood pressure was stable, it can be measured 1 ~ 2 times a week, and when there is fluctuation, it can be measured 1 ~ 2 times a day. In addition, it can monitor and record blood glucose levels before and after breakfast, lunch and dinner, and before bedtime. The blood lipids, glycosylated hemoglobin (Hemoglobin, HbA1c), urine glucose (Urine glucose, GLU), and urine ketone levels should be measured and recorded. It should instruct the patient’s family to use blood pressure monitor, blood glucose meter correctly.

Fifth, it should explain to patients and their families the importance of exercise for disease recovery, so that patients can better control blood sugar without metabolic disorders. It should instruct patients to choose appropriate exercise (walking, square dancing, swimming, cycling, etc.) and time 1 hour after meals according to their own conditions, and exercise 3 to 5 times a week. At the same time, it should make regular health assessments, and go out to exercise to prepare candy or other sugary substances to prevent hypoglycemia.

### Observation Index and Effect Evaluation Standard

The disease knowledge mastery (DKM) of patients was analyzed and compared after the intervention from 5 aspects to understand the mastery status, including diet, rehabilitation, nursing, medication guidance, and precautions. The total score of each aspect was 10 points, and the score was proportional to the degree of DKM.

The clinical efficacy was compared and graded into three levels. Grade I (excellent) meant that the symptoms of diabetes and hypertension were effectively controlled, the nursing effect was maintained for more than 1 year, the blood pressure level was less than 130/85 mmHg, the FPG level was 3.9 ~ 6.1 mmol/L, and the 2h PG was less than 10.0 mmol/L. Grade II (good) meant that diabetes and hypertension were basically controlled, but the effect was relatively unstable, and a phenomenon greater than the prescribed value occurs within 1 year. Grade III (poor) meant that blood pressure and blood sugar did not meet the specified value standards. The total effective rate (TER) = (level I + level II) / total number of cases × 100%.

Changes in blood sugar and blood pressure of patients were compared before and after nursing. PFG in the morning and 2h PG were detected by enzyme-linked immunosorbent assay (ELISA) using a Pumen automatic chemiluminescence analyzer (eCL8000, Shenzhen Hewlett Packard Medical Technology Co., Ltd.). PG level. The adult desktop sphygmomanometer from Shanghai Mediffin was adopted to monitor the patient’s DBP and SBP (the patient was instructed to rest quietly for more than 5 minutes before the test, do not smoke and drink coffee, and empty the bladder half an hour before). The average of the two monitoring results was calculated and recorded. If the difference in DBP and SBP was greater than 5 mmHg, then the average of the three monitoring results was calculated and recorded.

The mental states of patients before and after nursing were analyzed and compared. *Self-rating Depression Scale* (SDS) and *Self-Rating Anxiety Scale* (SAS) were used to evaluate the anxiety and depression of the two groups of patients before and after nursing. There were 20 items for each scale, each with 0 ~ 4 points. The critical value of the SDS scale was 53 points, 53 ~ 62 indicated the depression was mild, 63 ~ 72 indicated the depression was moderate, and > 72 indicated the depression was severe. The critical value of the SAS scale was 50 points, 50 - 59 indicated mild anxiety, 60 - 69 indicated moderate anxiety, and greater than 70 indicated severe anxiety.

### Statistical Analysis

The statistical data was processed by SPSS22.0. The count data was expressed in the form of N/%, using the χ^2^ test, and the measurement data was expressed as $\bar{\text{x}}\pm \text{s}$ , using the t test. *P* < 0.05 indicated that the difference was statistically significant.

## Results

### DKM

**Figure 1:**
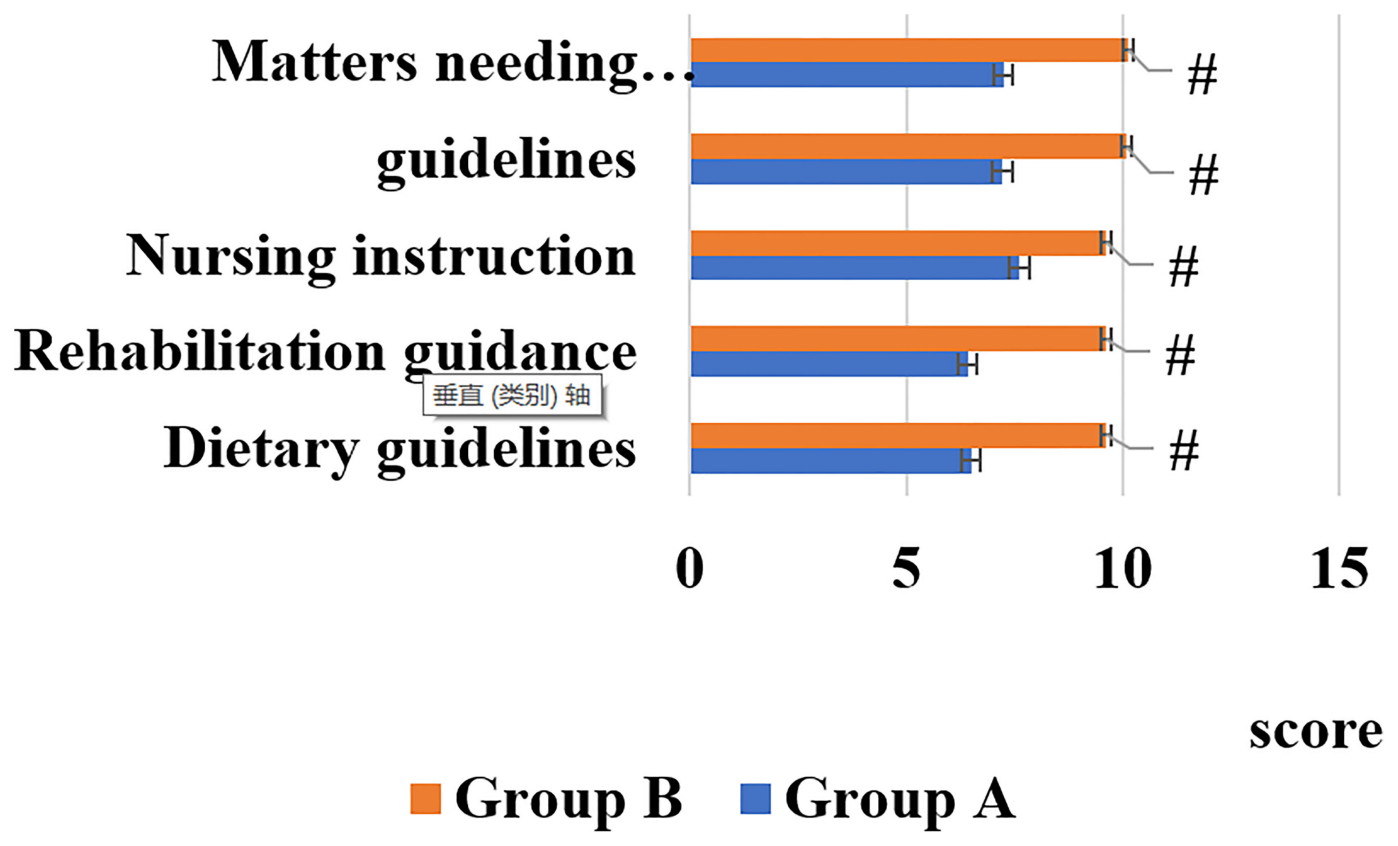
DKM of patients. [# indicated that compared with group A, the difference was statistically significant (*P* < 0.05)].

The DKM of two groups of patients was analyzed, as shown in **Figure 1**.

**Figure 1** illustrated that DKM of the patients in the group B was remarkably greater than that of the group A, showing statistically great difference (*P* < 0.05).

### Clinical Efficacy

**Figure 2:**
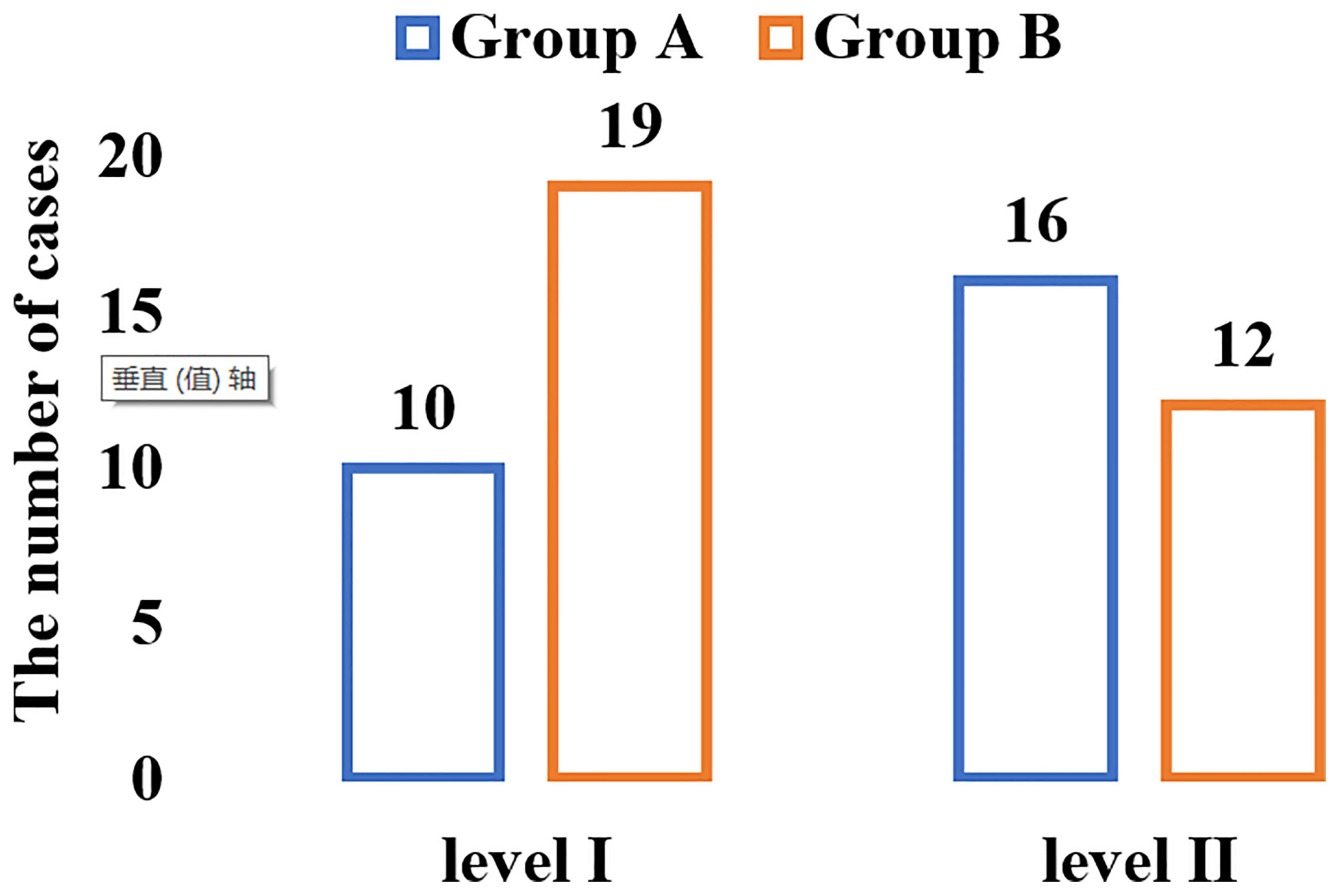
The results of the comparison of the clinical efficacy of the two groups of patients.

Comparison on the clinical efficacy of the two groups were shown in **Figure 2**.

As shown in **Figure 2**, there were 10 cases of grade I and 16 cases of grade II in group A; and 19 cases of grade I and 12 cases of grade II in group B. From the equation of TER = (grade I + grade II)/total number of cases × 100%, it can calculate the TER of the two groups of patients. The TER of patients in group B was 91.18%, which was significantly greater than that of group A (76.47%), and the difference was statistically significant (*P* < 0.05).

### Blood Sugar, Blood Pressure, and Rehabilitation Effect Before and After Nursing

**Figure 3:**
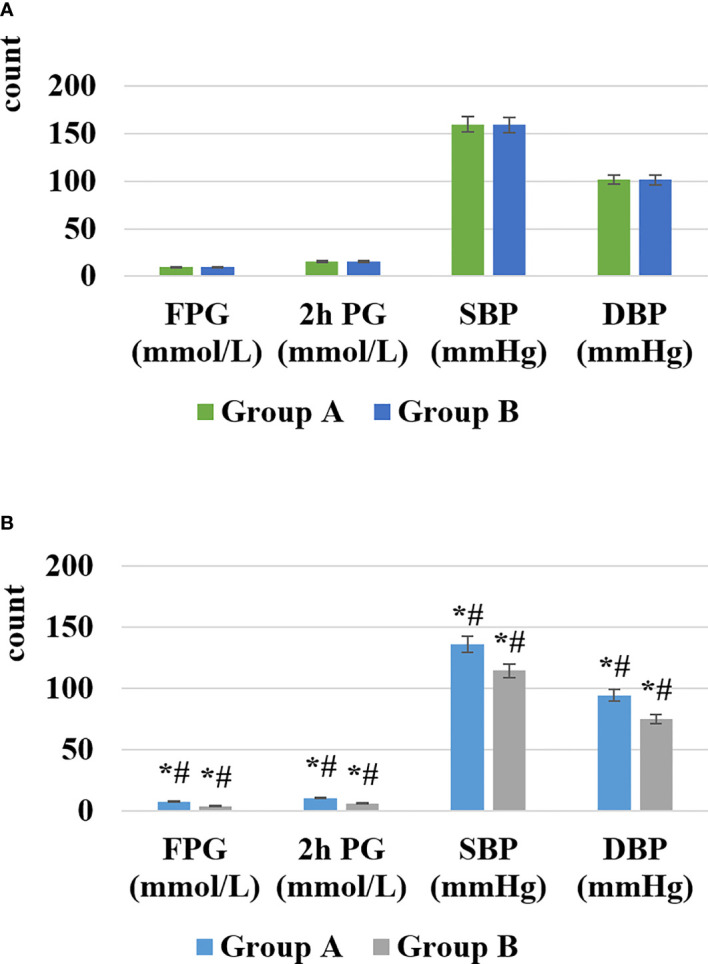
Comparison of blood glucose and blood pressure before and after nursing [**(A)** showed the change before nursing, **(B)** showed the change after nursing; *# represented the comparison with the case in the same group before nursing, the difference was statistically significant (P < 0.05)].

**Figure 4:**
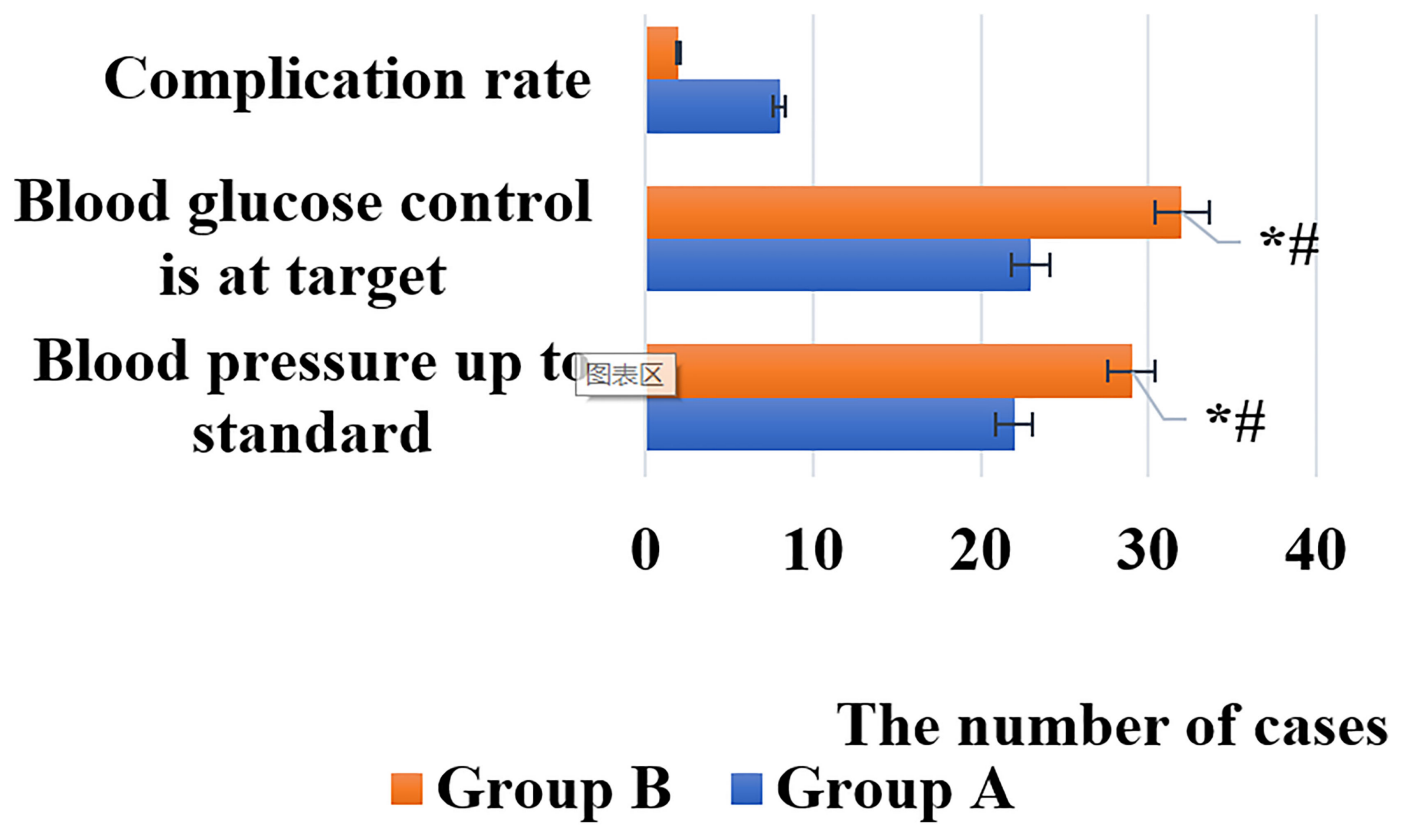
Comparison of rehabilitation effects between the two groups of patients [*# indicated that compared with group A, the difference was statistically significant (*P* < 0.05)].

The levels of FPG, 2h PG, SBP, DBP, and rehabilitation effect of the two groups of patients before and after nursing were shown in **Figures 3**, **4**.

As illustrated in **Figures 3**, **4**, after nursing, the levels of FPG, 2h PG, SBP, and DBP in the two groups of patients were significantly decreased, and those in group B were much lower than group A (*P* < 0.05). Similarly, the rehabilitation effect of patients in group B was significantly better than that in group A, and the difference was statistically significant (*P* < 0.05).

### Mental State Before and After Nursing

**Figure 5:**
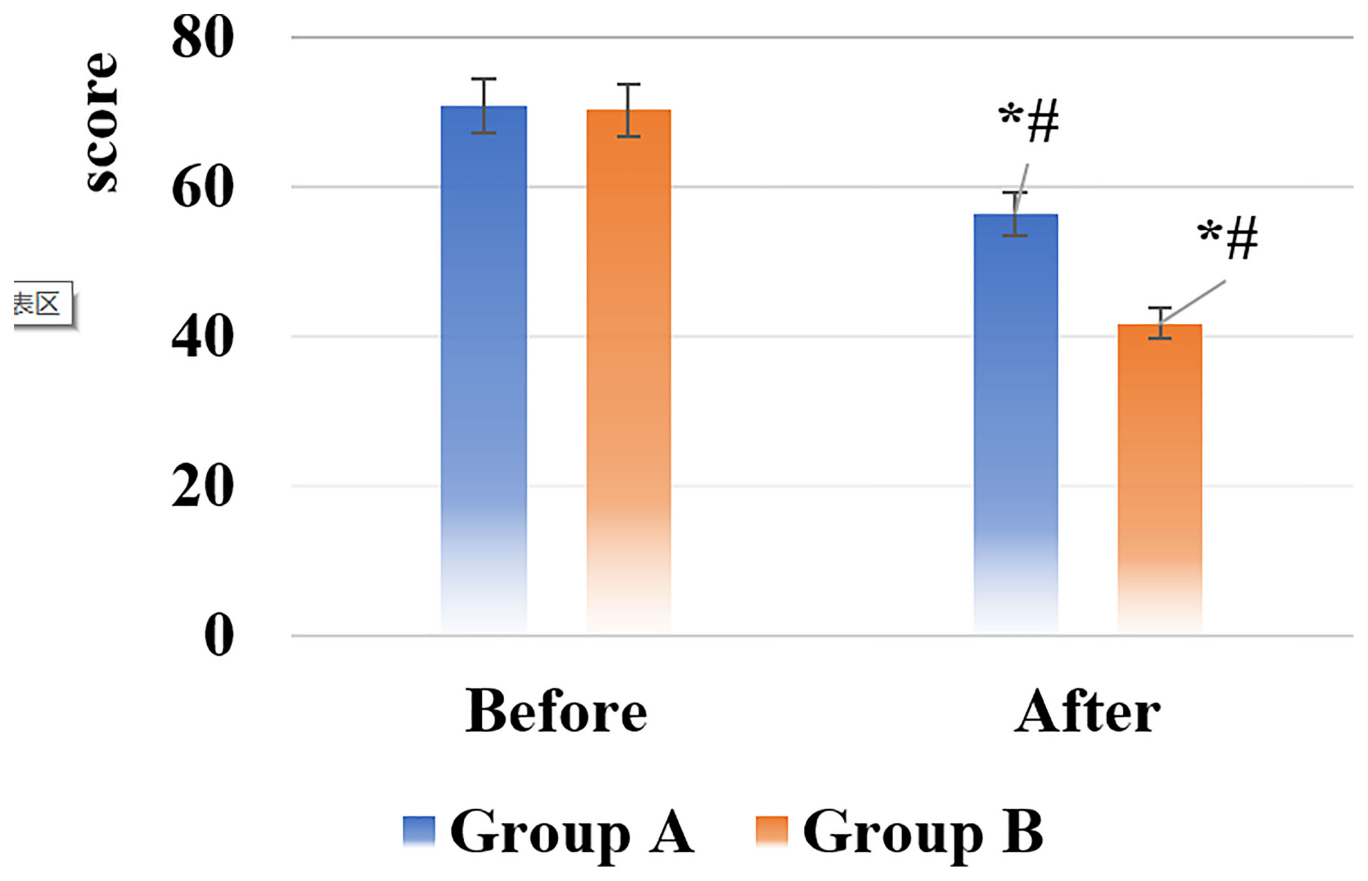
SDS score results of the two groups of patients before and after nursing [*# indicated that compared with the same group before nursing, the difference was statistically significant (*P* < 0.05)].

**Figure 6:**
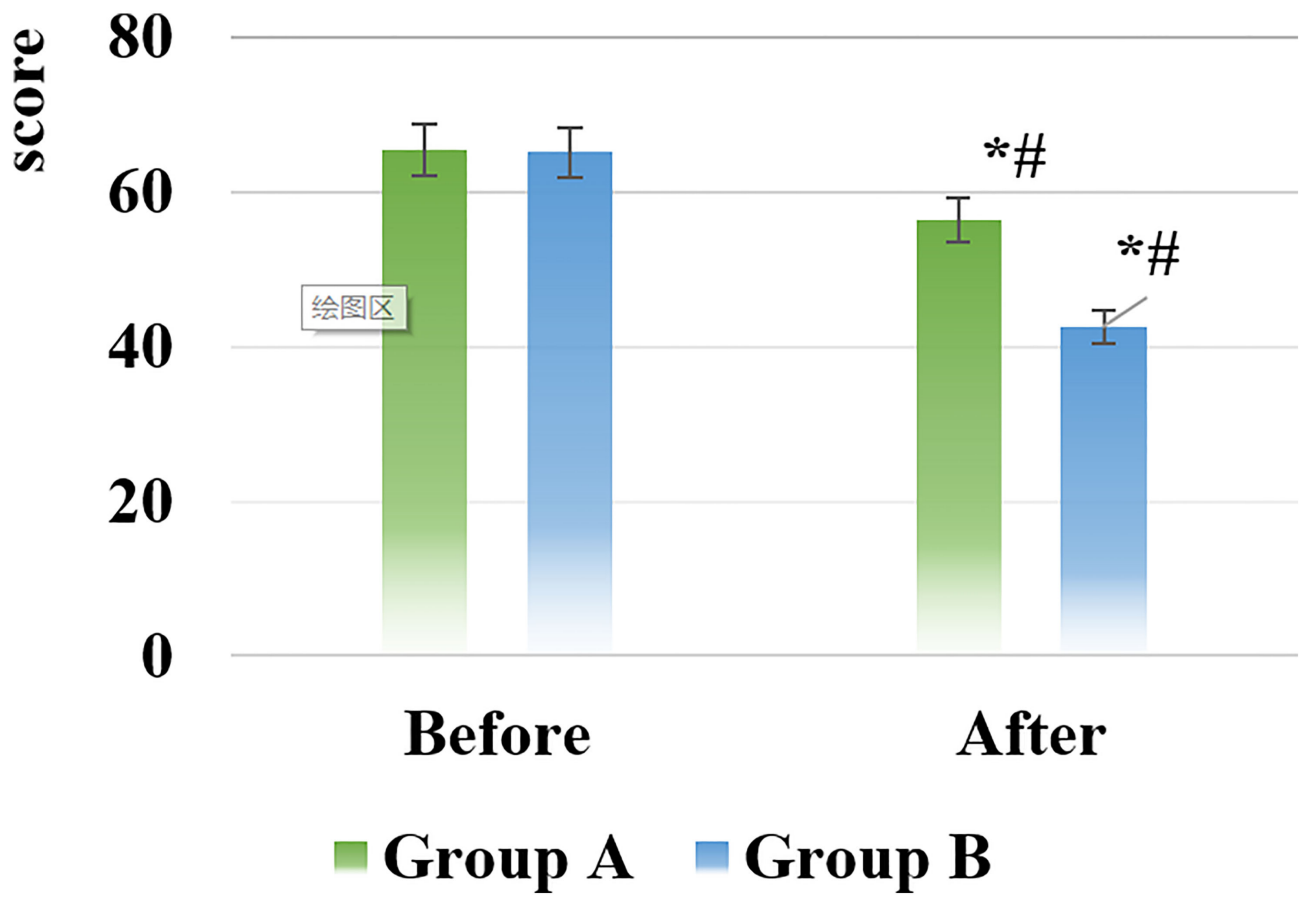
SAS score results of the two groups of patients before and after nursing [*# indicated that compared with the same group before nursing, the difference was statistically significant (*P* < 0.05)].

The scores of the Self-rating Depression Scale (SDS) and the Self-rating Anxiety Scale (SAS) of the patients in the two groups before and after nursing were shown in **Figures 5**, **6**.

After nursing, the scores of the SDS and SAS of all patients in groups A and B decreased, and those in the group B were significantly smaller than the scores in the group A, showing statistically visible differences (*P* < 0.05).

## Discussion

T2DM is a chronic disease caused by insufficient insulin secretion by pancreatic β-cells or caused by insensitivity of target cells to insulin. Its course is longer, the condition is more complicated, and there are many complications, the prognosis is poor, and it is easy to relapse (16). One of the very common complications of T2DM is hypertension, which is prone to cause cardiovascular and cerebrovascular diseases. The mortality and disability rate are relatively high. It is one of the important causes of death in diabetic patients and seriously threatens the patient’s life safety (17). Some scholars believe that the cause of hypertension associated with T2DM is that the increase in blood sugar inhibits the relaxation of vascular endothelium, stimulates the transcription of vascular smooth factor genes, and leads to the emergence of hypertension. In addition, the main risk factors for increased mortality from cardiovascular and cerebrovascular diseases are hypertension, hyperglycemia, and lipid metabolism disorders (18, 19). Therefore, increasing the regulation of blood sugar and blood pressure in patients with T2DM accompanied by hypertension is of high significance for the prevention of cardiovascular and cerebrovascular diseases.

At present, methods such as reducing blood sugar and blood lipids, adjusting body weight, and changing lifestyles are commonly used clinical methods to treat T2DM with hypertension. However, most patients have symptoms such as anxiety and depression due to long treatment time and repeated illnesses, resulting in poor control of blood sugar and blood pressure levels, resulting in a low treatment rate (20). Therefore, while actively treating patients, it is particularly important to carry out a scientific and reasonable nursing model. The implementation of individualized health education combined with a nursing model can enhance the patient’s enthusiasm for cooperating with treatment, eliminate bad emotions such as anxiety and depression, and effectively improve the patient’s quality of life.

After INHE, the levels of FPG, 2h PG, SBP, and DBP, and the scores of SDS and SAS in the two groups were decreased, and those in group B were significantly lower than those in group A (P < 0.05). The DKM and rehabilitation effect of patients in group B were significantly higher than those in group A (P < 0.05); and the TER of patients in group B was 91.18%, which was higher than 76.47% in group A (P < 0.05). It can be found that INHE can effectively improve the clinical treatment effect of patients, and has very good professionalism, pertinence, and effectiveness. In this work, the psychological and physiological characteristics of patients are combined for health education for them, which strengthened the patients’ awareness of the disease and better cooperates with clinical treatment. Combined with the individualized care model, effective psychological counseling is provided to patients, to eliminate negative emotions, and to promote patients’ mastery of the use of various commonly used drugs. Thereby, it can effectively prevent abnormal blood pressure and blood sugar of patients and improve the quality of life of patients.

## Conclusion

INHE was implemented for patients with T2DM complicated with hypertension to observe and compare DKM and rehabilitation effect of patients in the group B and the group A of patients. It was found that INHE can effectively improve the psychological cognition of patients with T2DM by hypertension, strengthen the control of blood pressure and blood sugar, and improve their quality of life. The innovation of this work lied in the combination of individualized nursing intervention and health education, which was more conducive to the rehabilitation of patients with diabetes and hypertension. However, the sample size was small, and it needed to further expand the research in the future, aiming to use more clinical experiments to verify the conclusion.

## Data Availability Statement

The original contributions presented in the study are included in the article/supplementary material. Further inquiries can be directed to the corresponding author.

## Ethics Statement

Ethical review and approval were not required for the study on human participants in accordance with the local legislation and institutional requirements. The patients/participants provided their written informed consent to participate in this study.

## Author Contributions

RL: conceptualization, data collection and analysis, and writing–original draft. WX: data collection and wrting–original draft. PY: visualization and data collection. LT: software and visualization. ZL: writing–editing and reviewing. XG: writing–editing and reviewing, and project administration. All authors contributed to the article and approved the submitted version.

## Funding

Scientific research funding program of the Second Affiliated Hospital of Chongqing Medical University (2021-13).

## Conflict of Interest

The authors declare that the research was conducted in the absence of any commercial or financial relationships that could be construed as a potential conflict of interest.

## Publisher’s Note

All claims expressed in this article are solely those of the authors and do not necessarily represent those of their affiliated organizations, or those of the publisher, the editors and the reviewers. Any product that may be evaluated in this article, or claim that may be made by its manufacturer, is not guaranteed or endorsed by the publisher.
